# Research progress of Claudin-low breast cancer

**DOI:** 10.3389/fonc.2023.1226118

**Published:** 2023-10-11

**Authors:** Chenglong Pan, Anqi Xu, Xiaoling Ma, Yanfei Yao, Youmei Zhao, Chunyan Wang, Ceshi Chen

**Affiliations:** ^1^Department of Pathology, First Affiliated Hospital of Kunming Medical University, Kunming, Yunnan, China; ^2^Kunming Medical University, Kunming, Yunnan, China; ^3^Department of Anesthesia, First Affiliated Hospital of Kunming Medical University, Kunming, Yunnan, China; ^4^Academy of Biomedical Engineering, Kunming Medical University, Kunming, Yunnan, China; ^5^The Third Affiliated Hospital, Kunming Medical University, Kunming, Yunnan, China

**Keywords:** breast cancer, Claudin-low, immunohistochemistry, mammary stem cells, epithelial-mesenchymal transformation

## Abstract

Claudin-low breast cancer (CLBC) is a subgroup of breast cancer discovered at the molecular level in 2007. Claudin is one of the primary proteins that make up tight junctions, and it plays crucial roles in anti-inflammatory and antitumor responses as well as the maintenance of water and electrolyte balance. Decreased expression of claudin results in the disruption of tight junction structures and the activation of downstream signaling pathways, which can lead to tumor formation. The origin of Claudin-low breast cancer is still in dispute. Claudin-low breast cancer is characterized by low expression of Claudin3, 4, 7, E-cadherin, and HER2 and high expression of Vimentin, Snai 1/2, Twist 1/2, Zeb 1/2, and ALDH1, as well as stem cell characteristics. The clinical onset of claudin-low breast cancer is at menopause age, and its histological grade is higher. This subtype of breast cancer is more likely to spread to lymph nodes than other subtypes. Claudin-low breast cancer is frequently accompanied by increased invasiveness and a poor prognosis. According to a clinical retrospective analysis, claudin-low breast cancer can achieve low pathological complete remission. At present, although several therapeutic targets of claudin-low breast cancer have been identified, the effective treatment remains in basic research stages, and no animal studies or clinical trials have been designed. The origin, molecular biological characteristics, pathological characteristics, treatment, and prognosis of CLBC are extensively discussed in this article. This will contribute to a comprehensive understanding of CLBC and serve as the foundation for the individualization of breast cancer treatment.

## 1 Introduction

Breast cancer is one of the three most common cancers in the world. Hierarchical cluster analysis of the genes that vary more between tumors than between repeated samples of the same tumor has revealed the existence of four major breast cancer intrinsic subtypes (luminal A, luminal B, HER2-enriched, and basal-like), as well as a normal breast-like group (1, 2). In 2007, Herschkowitz et al. (3) found a breast cancer subtype called the claudin-low subtype upon the examination of murine and human breast tumors. This newly discovered subtype of breast cancer has features of low expression of tight junction proteins and adhesion proteins (Claudin3, 4, 7, and E-cadherin) and luminal markers but high expression of basal-related genes and lymphocyte- and endothelial cell-related markers. Recently, scientists have paid more attention to this intrinsic molecular subtype of breast cancer, which is known as claudin-low breast cancer (CLBC). A comprehensive analysis of CLBC will help us to better understand this intrinsic subtype of breast cancer.

## 2 Structure and function of claudins

**Figure 1:**
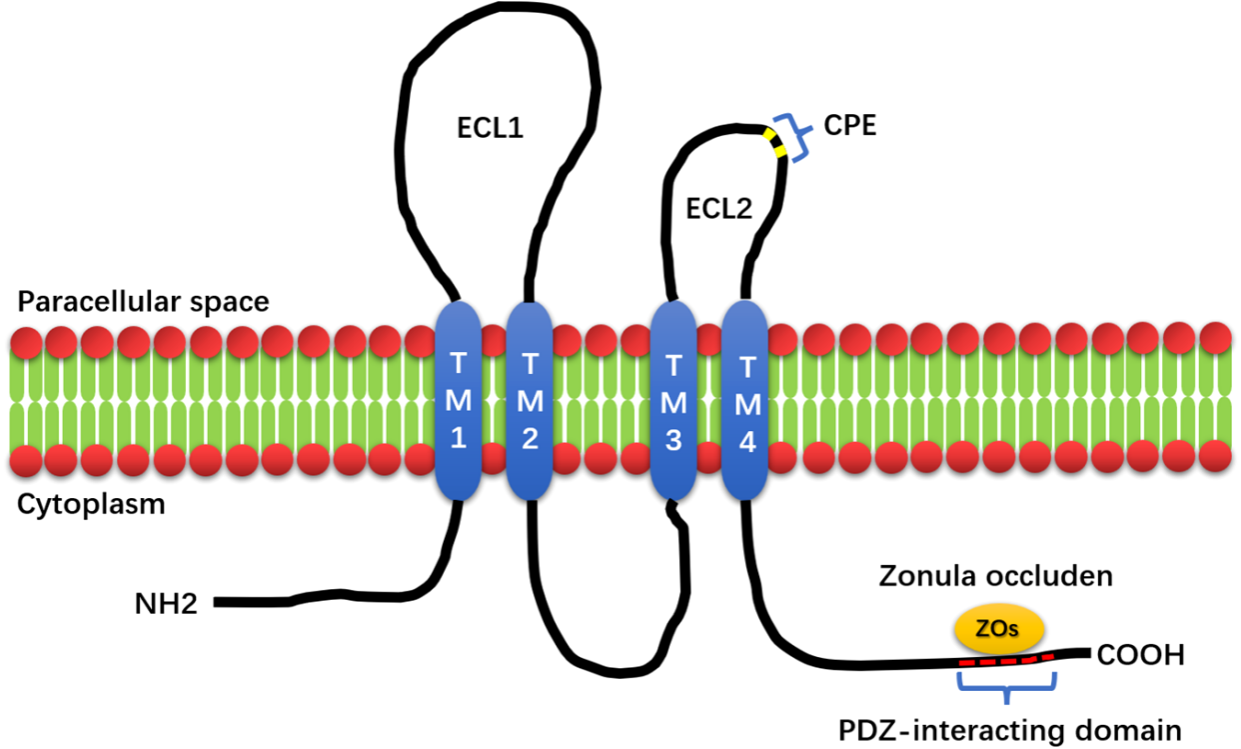
The structure of Claudin. The structure of Claudin mainly consists of four transmembrane structures (TM1, 2, 3, and 4), two extracellular loops, and one intracellular loop (ECL1, ECL2). The small extracellular loop contains the *Clostridium perfringens* enterotoxin-binding receptor (CPE), and the PDZ-interacting domain at the C-terminus of Claudin is capable of binding to Zonula occluden (Zos) to maintain cell homeostasis.

Furuse and Tsukita first discovered claudin in 1998. Claudin derives from the Latin word ‘claudere’, which means to close (4, 5). Claudins (CLDN) are cell–cell adhesion proteins that are expressed at tight junctions (TJs), which are the most prevalent apical cell–cell adhesions (6). Tight junctions, together with adherens junctions and desmosomes, form the apical junctional complex in epithelial and endothelial cellular sheets. Adherens junctions and desmosomes are responsible for the mechanical adhesion between adjacent cells, whereas tight junctions are essential for the tight sealing of the cellular sheets, thus controlling paracellular ion flux and therefore maintaining tissue homeostasis (4, 7, 8). The tight junction proteins are diverse and include occludins, claudins, tricellulin, cingulin, and junctional adhesion molecules (JAMs). These proteins interact within themselves and with the cellular cytoskeleton to form a complex architecture. Among these TJ proteins, claudins are key proteins that act as both pores and barriers, aiding the paracellular pathway between epithelial cells (9). In mammals, it is composed of 27 members, and specific CLDN combinations are expressed in specific cell and tissue types. Claudins can be divided into classical claudins and nonclassical claudins according to the homology of the claudin sequence and its function (10). Claudin has four transmembrane proteins; in mammals, 27 members are between 20 and 34 kDa in size (11). Claudin has four transmembrane helices, and its amino- and carboxy-termini penetrate deep into the cytoplasm. The N-terminal domain of Claudins is relatively short, contain 7 amino acid sequences followed by a large extracellular loop (ECL1) of approximately 50 amino acid sequences. A short inner loop is separated by a small extracellular loop (ECL2) of approximately 25 amino acid residues. The main role of ECL1 is paracellular transport with selective ion permeability. The role of ECL2 is to participate in the interaction between Claudins. ECL2 of Claudin3 and Claudin4 contains binding receptors for *Clostridium perfringens* enterotoxin (CPE) (5, 10, 12–14). Studies have found that CPE can be used as a target for the development of claudin-targeted drugs (15–17). The localization and function of claudin are also regulated by the phosphorylation of the C-terminus, a target of serine, threonine, and tyrosine kinases. Claudin regulates functional changes in TJ proteins through posttranslational modifications, such as phosphorylation, ubiquitination, palmitoylation, and glycosylation, to regulate claudin conformation, stability, transport, and function (10). The C-terminus of claudins contains a PDZ-binding domain, for signal transduction. Claudin binds to Zonula occludens (ZO1, ZO2, and ZO3 (18)), Pals1-associated tight junction protein (PATJ) (19) and Multi-PDZ domain protein 1 (MUPP1) (20) through a PDZ-interacting domain, which plays an important role in cell function. ZO-1, ZO-2, and ZO-3 are TJ-related proteins (21–23). ZO-1 and ZO-2 are key proteins for cell junction assembly and permeability, respectively (24, 25). When ZO-1 and ZO-2 are missing, cells cannot assemble TJs (26, 27). The structure diagram of claudin is shown in **Figure 1**. The PDZ domain-binding motif located at the C-terminal end of claudin cytoplasm can directly interact with ZO family proteins, which plays important roles in many cellular processes.

Whether different claudin proteins copolymerize to form tight junction chains as heteropolymers, and whether claudin interacts in a homogeneous manner, between two molecules of the same claudin member, or between two different claudin members remains unclear. According to a previous study, different claudin members can interact within and between tight junction strands, but these combinations were restricted to specific combinations of isoforms (28).

Claudin is widely expressed in many different tissues. The majority of claudin expression occurs in barrier-forming epithelial cells and endothelial cells. Not all claudins are expressed in all tissues at the same time, and these differences in how claudins are expressed control the functions of cells, especially paracellular barrier functions. Claudins play a key role in regulating the paracellular permeability selectivity. The overexpression of Claudin affects the resistance and permeability of epithelial cells to different ions, and ECL1 plays an important role in the selectivity of charge (29, 30). Various mouse models have shown that Claudin plays an important role in cellular barriers. For example, claudin-1-deficient mice are dehydrated due to increased cell permeability, leading to rapid death (31). Claudin shows abnormal expression in several cancers, which is related to the occurrence and progression of cancer. Claudin-1 is not expressed in breast cancer and colon cancer, which is related to the decomposition of TJs in tumor development (32, 33). Claudin is a double-edged sword. The loss or low expression of Claudin in some tumors is related to the progression, invasion and metastasis of cancer, such as gastric cancer (34), esophageal squamous cell carcinoma (35) and colorectal cancer (36). Claudin has the opposite effect in some cancers. Claudin can directly or indirectly activate various signal pathways or proteases, and promote the occurrence of tumors. Therefore, it has been observed that Claudin is highly expressed in some tumors. Claudin-3 and -4 have been found to be expressed in many types of tumors, and some studies have shown that the overexpression of Claudin in tumors is related to tumor growth and invasion (16, 37), such as ovarian cancer (38, 39), glioma (40) and pancreatic cancer (41, 42). The role of Claudin in cancer stem cell biology through the WNT pathway has attracted increasing attention. Claudin-1 and -2 transcription is regulated by WNT signaling, which is known to regulate the β-Catenin-T-cell factor/lymphoid enhancer-binding factor (TCF/LEF) signaling pathway and stemness (43, 44).

## 3 Definition of claudin-low breast cancer

CLBC was originally defined by a gene expression signature, which represents the low expression of claudin and cell-cell adhesion-related genes, including claudin 3, 4, and 7, and the high expression of EMT-related genes and breast cancer stem cell-related genes (45). Defining CLBC by genome is the foundation for most researchers to study CLBC. In addition, some researchers defined CLBC by immunohistochemistry. Most scholars define the low expression of claudins 3, 4, and 7 in the tumor cell membrane as CLBC. However, unlike HER2 in immunohistochemical staining, there is no unified standard to define the low expression and high expression of claudin in the interpretation of Claudin immunohistochemical (IHC) staining. Some scholars have multiplied the staining intensity and staining degree of claudin in tumor cells and delineated the corresponding boundary to define the high and low expression of claudin (46–50). Other researchers defined CLBC when less than 50% of tumor cells express claudins 3 and 4 and less than 5% of tumor cells express claudin 7 (51). However, some scholars have recently put forward a new view that Claudin-low is a biological characteristic that describes breast cancer and is not equivalent to the inherent molecular classification of breast cancer (52). Therefore, we believe that the definition of Claudin-low breast cancer should be a type of breast tumor characterized by low expression or absence of epithelial adhesion proteins (Claudin3, Claudin4, Claudin7 and E-cadherin, etc.). Compared with the intrinsic molecular classification of breast cancer, CLBC has its own unique characteristics in clinical pathology, molecular biology and other aspects.

## 4 The origin of claudin-low breast cancer

The origin of CLBC has been disputed. At present, there are three main theories about the origin of CLBC. Some scholars have found that CLBC has features of tumor-initiating cells or stem cells. They speculated that the intrinsic subtype of breast cancer may reflect different stages of epithelial development. CLBC represents the most primitive tumors that are similar to mammary stem cells and may originate from mammary stem cells (MaSCs) (53–55). Previous studies have found that the active form of RAS and various cytokines, including transforming growth factor-β (TGF-β), promote epithelial-mesenchymal transformation (EMT), which transforms breast epithelial cells into malignant cells, which have all the characteristics of CLBC (56–59). Many factors regulate the transformation of luminal epithelium into CLBC through EMT. For example, oncogenic RAS signaling drives the occurrence and progression of triple-negative breast cancer from the luminal epithelium (60). Absence of the Notch signaling regulators Lunatic Fringe (Lfng) and p53 leads to CLBC (61). TbL1 interacts with *ZEB1*, which inhibits the activation of the E-cadherin (CDH-1) gene, activates the *ZEB1* gene promoter, and promotes the transformation of mammary epithelial cells to CLBC (62). There is evidence that, under the influence of genetic mutations or environmental conditions, reactivation or dedifferentiation of luminal epithelial cells induces and accelerates the formation of more aggressive mammary tumors. Furthermore, the deletion of p53 can lead to clonal proliferation of the luminal epithelium, which promotes the development of breast tumors and the acquisition of MaSC characteristics, resulting in the formation of CLBC (63–65). In recent years, some scholars have deleted the *Pten* gene in the mouse mammary epithelium and induced the p53-R270H mutation, leading to CLBC (66). Other scholars have found that the synergistic effect of MET and p53 deletion can induce CLBC (67). However, according to the study by Pommier et al. (68), CLBC shows remarkable diversity. Based on the analysis of genetics, gene methylation, and gene expression, they found that CLBC arises from three subgroups, two of which are related to luminal subtype breast cancer and basal-like subtype breast cancer, which are transformed by activating the EMT process during tumor development. The third subgroup is closely related to normal human breast stem cells (MaSCs), showing genome distortion and a low frequency of the TP53 mutation. In short, the origin of CLBC is a complicated process.

According to previous studies, claudin-low breast cancer has always been considered to fall into the category of triple-negative breast cancer. According to Pommier et al. (68), claudin-low breast cancer may originate from various stages of breast cancer development, and this is likely the best explanation for the high expression of breast cancer stem cell markers and EMT-related markers in claudin-low breast cancer as well as its overlap in gene and immune expression with luminal A/B, HER2 overexpression, and triple-negative breast cancer. The Shanghai Cancer Center of Fudan University (FUSCC) used androgen receptor (AR), CD8, FOXC1, and DCLK1 as immunohistochemical markers and classified TNBCs into five subtypes based on the staining results: (a) IHC‐based luminal androgen receptor (IHC‐LAR; AR+), (b) IHC‐based immunomodulatory (IHC‐IM; AR−, CD8+), (c) IHC‐based basal‐like immune‐suppressed (IHC‐BLIS; AR−, CD8−, FOXC1+), (d) IHC‐based mesenchymal (IHC‐MES; AR−, CD8−, FOXC1−, DCLK1+), and (e) IHC‐based unclassifiable (AR−, CD8−, FOXC1−, DCLK1−) (69). Currently, there is no relevant research showing the relationship between claudin-low and FUSCC triple-negative breast cancer staging. Since claudin breast cancer is positive for EMT and stem cell-related immune markers, we believe it is more related to MES.

## 5 The mechanism of Claudin-low carcinogenesis

Claudin is highly expressed in normal tissues. Although the expression of Claudin varies across tumors, it is decreased in many tumors, indicating that Claudin can inhibit the function of tumors. The increased expression of Claudin significantly inhibits the proliferation of tumor cells and promotes EMT, migration and invasion of tumors. Low expression of claudins is associated with advanced disease, metastasis, and a poor prognosis (70). At present, the mechanism by which Claudin inhibits tumor occurrence is not clear, but some scholars have proposed the following three hypotheses: (A) Claudin is able to prevent microorganisms, toxins, and growth factors from passing through the paracellular channel, which are common oncogenic factors. In the absence of Claudin, these oncogenic factors enter the body and are likely to induce tumors (71–73). (B) Claudin directly binds or utilizes other scaffold proteins [mainly zonula occludens (ZOs)] to indirectly bind some key factors of signaling pathways, such as yes-associated protein/transcriptional coactivator (YAP/TAZ), pyruvate dehydrogenase kinase 1 (PDK-1), and β-catenin, which are all well-known carcinogenic factors. Claudin keeps them on the cell membrane and blocks carcinogenic signaling pathways, such as the PDK-AKT and YAP/TAZ-TEAD pathways (74–77). Reduced expression of ZO-1 in breast cancer destroys the structural integrity of the tight junction, leading to a loss of cell-cell adhesion. MUPP-1 expression is reduced in patients with poor prognosis and increased tumor grade. MUPP-1, like ZO-1, can be used as a crosslinker between claudin and tight-linked chains and tight-linked JAM oligomers, and other integral membrane proteins can be recruited into tight-linked claudin via MUPP-1. Studies have found that the transcription levels of ZO-1 and MUPP-1 in metastatic breast cancer are significantly reduced. The higher the expression of ZO-1 in breast cancer, the worse the prognosis of breast cancer patients (78). Normal tissues at the tight junction of mammary epithelial cells showed strong ZO-1 staining, and approximately 70% of breast cancers were found to have reduced or lost ZO-1. ZO-1 staining was positively correlated with tumor differentiation, and the reduction in ZO-1 staining was closely related to the reduction in E-cadherin staining. Therefore, ZO-1 may be directly involved in the malignant progression of breast cancer (79). A decrease in ZO-2 expression has also been observed in most breast cancers (80). (C) The basement membrane Claudin was found to colocalize with integrin β1 and form a protein complex to maintain epithelial cell attachment and inhibit cell proliferation. If claudin is depleted or expressed at low levels, epithelial cells become destabilized and proliferative (81, 82).

Apart from causing cancer in the above three aspects, Claudin also inhibits EMT. EMT is associated with tumorigenesis, progression and fibrosis (83). Transforming growth factor beta 1 (TGF-β1) has been shown to induce EMT during various stages of embryogenesis and progressive disease. Medici et al. (84) found that claudin protein expression was missing when TGF-β was used to induce EMT. SMAD3 and SMAD4 interact with SNAIL1, a transcriptional repressor and EMT promoter, and form a complex (SNAIL1-SMAD3/4) that targets the gene promoter of CAR, a tight junction protein, and E-cadherin. SNAIL1 and SMAD3/4 act as corepressors of the CAR, occludin, claudin-3, and E-cadherin promoters. In contrast, the combined silencing of SNAIL1 and SMAD4 with siRNA promoted the transcription of CAR and occludin during EMT (85). These studies indicate that Claudin is inhibited during EMT. Studies have found that the deletion of Claudin3 or Claudin4 leads to activation of the PI3K pathway, which is manifested as increased Akt phosphorylation, increased PIP3 content and PI3K activity, as well as up regulation of mRNA and protein levels of the transcription factor Twist. Claudin3 and Claudin4 maintain the epithelial phenotype, and their deletion promotes EMT (86).

Tumor metastasis is an important step in the process of tumor progression. The interaction between cancer cells and endothelial cells is the key for the distant metastasis of tumor cells. In the process of tumor metastasis, tightly connected Claudin participates in the defense of tumor cells, and tightly connected Claudin is the barrier of paracellular channels for epithelial and endothelial cell material exchange (87). Researchers believe that the loss of adhesion of tumor epithelial cells is a necessary condition for tumor invasion and metastasis. The expression of claudin in human cancer may decrease or increase in a tissue-specific manner. Claudin is responsible for the structural and functional integrity of the tight junction of the epithelial cell layer. The decrease or loss of claudin expression is accompanied by cell-cell adhesion and polarity damage. Tumorigenesis is accompanied by the destruction of tight junctions, a process that plays an important role in the loss of adhesion and the enhancement of invasiveness in tumor cells. The loss of Claudins and other tight junction proteins in cancer is considered a mechanism of cell adhesion loss and an important step in metastasis (88). Claudin constructs a complete biological system based on the function of the cell-side barrier. Generally, these claudin functional deficits are associated with water imbalance, inflammation, cancer, and brain disease, depending on the tissues and organs involved (31, 74, 89–92). Many studies have found that Claudin is expressed at low levels in many breast cancer tissues (93–95).

The feedback pathway of ZEB1/miR-200a promotes the invasion and metastasis of breast cancer cells (96, 97). It was found that miR-200a can regulate YAP1 in the HIPPO pathway and promote the survival and metastasis of CLBC (98). The ectopic expression of miR-200a can weaken the migration and invasion of CLBC by reducing the expression level of *ELK3* mRNA (99). Compared with other subtypes of breast cancer, CLBC has the characteristics of a vascular system and endothelium and has higher vascular permeability, which promotes the metastasis of CLBC (100).

**Figure 2:**
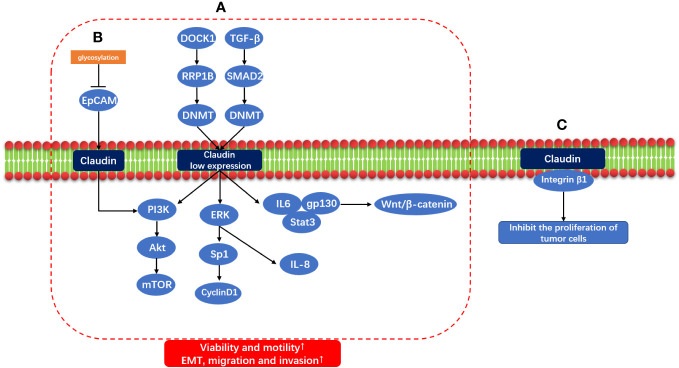
Claudin and Claudin low-expression carcinogenic signal pathway. **(A)** DOCK1/RRP1B/DNMT and TGF-β/SMAD2/DNMT induce Claudin low expression through methylation, and Claudin low expression through IL6/gp130/Stat3, Wnt/β-Catenin signal pathway; ERK/Sp1/CyclinD1 and ERK/IL8; PI3K/AKt/mTOR promotes EMT, migration and invasion of tumor. **(B)** Glycosylation of EPCAM was inhibited, and PI3K/AKt/mTOR was activated by combining with Claudin to promote EMT. **(C)** Claudin and Integrin β1 also inhibit the proliferation of tumor cells.

It has been proved that changes of protein glycosylation is involved in the regulation of EMT process of cancer cells (101). Claudin and low expression of Claudin play a role in carcinogenesis through multiple signaling pathways. Dedicator of cytokinesis 1 (DOCK1) down-regulates Claudin through ribosomal RNA processing 1B (RRP1B)/DNA methyltransferase (DNMT) to promote the growth and migration of breast cancer cells (102); Claudin through down-regulates the transforming growth factor-β(TGF-β)/Smad2/DNMT to promote EMT, metastasis and invasion of breast cancer cells (103). Low expression of Claudin promotes EMT by activating PI3K/AKt/mTOR, and regulates ERK/Sp1/CyclinD1 and ERK/IL-8 to promote the proliferation, migration and invasion of breast cancer cells (104). Claudin dysregulation promotes tumorigenesis by upregulating gp130/IL6/Stat3 signaling and activating the Wnt/β-catenin signaling pathway (74). Epithelial cell adhesion molecule (EpCAM) is a homophilic type I transmembrane glycoprotein belonging to the small GA733 protein family, EpCAM functions not only in physiological processes but also participates in the development and progression of cancer (105). The glycosylation of EpCAM is inhibited, which increases the expression of EpCAM in breast cancer. At the same time, the combination of Claudin and EpCAM increases, promotes the phosphorylation of PI3K/Akt and p38, promotes the activation of MAPK and PI3K/Akt pathways, promotes the process of EMT, reduces Apoptotic ability of breast cancer cells, promoting cell proliferation (106). In addition, Claudin and Integrin β1 inhibit the proliferation of tumor cells. If Claudin is missing, the combination of Claudin and Integrin β1 will be reduced, which will promote the tumor (81). The mechanism of claudin and claudin low-expression carcinogenic signal pathway is shown in **Figure 2**.

## 6 Molecular biological characteristics of claudin-low breast cancer

Currently, the following cell lines are used to study claudin-low breast cancer: MDA-MB157, MDA-MB436, BT549, MDA-MB231, HBL100, SUM159PT, Hs578T, MDA-MB435, and SUM1315. Some studies have extracted two cell lines, RM11A and RJ348, from breast tumors developed in MTB-IGFIR transgenic mice, which have the histological characteristics and gene expression pattern of claudin-low breast cancer and have been reinjected into the mouse breast fat pad to obtain high tumorigenicity (107). Fougner et al. (108) found that claudin-low-like MPA/DMBA-induced mouse mammary tumors represent a transcriptionally accurate model for human claudin-low breast cancer.

### 6.1 Gene expression characteristics of claudin-low breast cancer

CLBC has the features of EMT and stem cells (109). Taube et al. (110) showed that tumor cells gain a certain degree of invasiveness and metastasize through EMT; at the same time, they also gain the potential for self-renewal, proliferating to form a macroscopically metastatic large cell population. Studies have shown that TGF-β induces EMT through the TGF-β/SMAD/LEF/PDGF pathway and the MAPK pathway (111). In addition, the homeobox proteins Goosecoid and the zinc finger proteins SNAIL and TWIST can also induce EMT. Another study found that miR-200 expression decreases during EMT (112). Surprisingly, Jones et al. (113) re-expressed miR-200c in murine claudin-low breast tumor cells to inhibit tumor cell proliferation and growth. Therefore, they believe that the expression of miR-200c can inhibit the growth of claudin-low breast tumor cells.

In addition, CLBC has a high degree of immune cell infiltration and high expression of T and B lymphoid cell markers (such as CD14 and CD79a) (45, 114, 115). Some scholars have compared CLBC with non-claudin-low breast cancer and found that CLBC has few gene mutations, low genetic instability, a low frequency of TP53 and PIK3CA mutations, and minimal MYC and MDM4 gain (52). Through Gene Ontology (GO) analysis, 165 genes were found to be differentially expressed in CLBC, among which 69 were upregulated and 64 downregulated. They analyzed 193 pathways and found that the inflammatory IL-13 signaling pathway was significantly enriched. Among them, five upregulated genes (IL6, CXCL8, VEGF-C, NRF1, and EREG) were mapped as hubs and may play an important role in CLBC (116). The comparison of Claudin-low breast cancer and other subtypes of breast cancer in immunohistochemistry and gene phenotype is shown in **Table 1**.

**Table 1:** Immunohistochemical and gene comparison of Claudin-low breast cancer and other subtypes of breast cancer.

IHC/Gene	Claudin-low	Luminal A/B	HER 2	TNBC
Claudin3	Negative	Low expression/Negative	Low expression/Negative	Low expression/Negative
Claudin4	Negative	Low expression/Negative	Low expression/Negative	Low expression/Negative
Claudin7	Negative	Low expression/Negative	Low expression/Negative	Low expression/Negative
E-cadherin	Negative	Low expression/Negative	Low expression/Negative	Low expression/Negative
ER	Negative	Low expression/Negative	Negative	Negative
PR	Negative	Low expression/Negative	Negative	Negative
HER2	Negative	Low expression/Negative	Hight expression	Negative
CK5	Negative	Low expression/Negative	Low expression/Negative	Low expression/Negative
CK8	Negative	Low expression/Negative	Low expression/Negative	Low expression/Negative
CK14	Negative	Low expression/Negative	Low expression/Negative	Low expression/Negative
CK17	Negative	Low expression/Negative	Low expression/Negative	Low expression/Negative
CK18	Negative	Low expression/Negative	Low expression/Negative	Low expression/Negative
CK19	Negative	Low expression/Negative	Low expression/Negative	Low expression/Negative
Vimentin	Hight expression	Negative	Negative	Negative
SNAI1	Hight expression	Negative	Negative	Negative
SNAI2	Hight expression	Negative	Negative	Negative
TWIST1	Hight expression	Negative	Negative	Negative
TWIST2	Hight expression	Negative	Negative	Negative
ZEB1	Hight expression	Negative	Negative	Negative
ZEB2	Hight expression	Negative	Negative	Negative
ALDH1	Hight expression	Negative	Negative	Negative
CD44	Hight expression	Negative	Negative	Low expression
PD-L1	Hight expression	—	—	—
Caveolin-1	Hight expression	—	—	—
Galectin-1	Hight expression	—	—	—
SMYD3	Hight expression	—	—	—
CD24	Negative	Negative	Hight expression	Low expression
Ki-67	Low proliferation index	—	—	—
GATA3	Negative	Low expression	Negative	Negative
EPCAM	Negative	Negative	Low expression	Negative
CD10	Low expression	Negative	Negative	Negative

"-" means that these marker are not related to literature reports.

### 6.2 Claudin-low breast cancer is related to the MAPK pathway

Several studies have shown that RAS/MAPK kinases are highly activated in breast cancer (60, 68, 108). The dataset from the Molecular Taxonomy of Breast Cancer International Consortium (METABRIC) and TCGA cohorts was studied, and RAS signals were found to be highly expressed in CLBC. The GDSC database was also used to observe the IC50 of three MEK inhibitors (trametinib, selumetinib, and refametinib) in various subtypes of breast cancer, and CLBC was sensitive to three MEK inhibitors compared with other subtypes. This finding indicates that CLBC is driven by the MAPK pathway. Unfortunately, these findings were only performed on the genetic claudin-low breast cancer cell line and the corresponding mouse model, and clinical trial studies are lacking.

## 7 Clinicopathological characteristics of claudin-low breast cancer

### 7.1 Patient age

At present, there is still controversy about the age of CLBC. Some scholars have found that patients with CLBC are younger than patients with other subtypes (114, 115, 117, 118). However, Xu et al. (119) found that the age of CLBC patients is not significantly different from other subtypes, which may be related to their small sample size. Fougner et al. (52) found that the onset age of patients with negative ER, PR and HER in CLCB is younger than that of patients without negative ER, PR and HER. However, when they compared the CLBC and non-CLBC groups, the ages of the two groups of patients were similar.

### 7.2 Tumor characteristics: tumor size, histological grade, and lymph node metastasis

The research results of Sabatier et al. (114) and Xu et al. (119) were consistent, in that CLBC was mainly larger than 2 cm (62%, 61.9%). In the tumor group larger than 2 cm, the proportion of CLBC was lower than that of the HER2-enriched subtype and the basal-like subtype and higher than that of the luminal subtype and the normal-like subtype. They also found that the proportion of histological grade 3 was higher than that of other subtypes (56%, 71.4%), except for the base-like subtype (85%, 56.6%). The rate of lymph node metastasis (46%, 38.1%) was higher than that of the base-like subtype (35%, 25%) and luminal A subtype (54%, 26.8%). This indicates that CLBC has a larger tumor diameter, a higher histological grade, and a higher rate of axillary lymph node metastasis. This may be related to the low expression of adhesion-related proteins in CLBC, which makes it easier to transfer.

### 7.3 Histopathology

Prat et al. (45) found that approximately 50% (8/16) of CLBCs are metaplastic carcinomas,

and Sabatier et al. (114) found that CLBCs are mainly nonspecialized breast cancer (77.7%), metaplastic carcinoma (71.4%), and medullary carcinoma (24%). Some scholars have also found that the histological types of CLBC are mainly invasive lobular carcinoma, metaplastic carcinoma, and medullary carcinoma (120). In short, the histological types of CLBC are mainly poorly adherent carcinomas, metaplastic carcinomas, and medullary carcinomas. Both CLBC and lobular carcinoma have down-regulation of E-cadherin, but CLBC does not always show the histological characteristics of lobular carcinoma. This may be along with the down-regulation of E-cadherin and other molecular changes, CLBC is more prone to ductal carcinoma. Characteristically, in contrast to lobular carcinoma, downregulation of E-cadherin in CLBC is associated with epigenetic or posttranscriptional dysregulation (121).

### 7.4 Immunohistochemistry

CLBC has low expression of tight junction proteins and adhesion proteins (Claudin3, 4, 7 and E-cadherin), and it also has low expression of cytokeratin (CK5, 8, 14, 17, 18, 19, and 24), HER-2 and luminal-related markers (45, 115). These features are similar to the basal-like subtype of breast cancer. CLBC has low expression of proliferation genes and Ki-67, which is inconsistent with the basal-like subtype of breast cancer (45, 115). Compared with other subtypes of breast cancer, CLBC has high expression of EMT-related markers (such as Vimentin, SNAI1 and SNAI2, TWIST1 and TWIST2, and ZEB1 and ZEB2) (114) and breast cancer stem cells/tumors with the initial cell markers ALDH1 and CD44^hi/^CD24^-/low^ (115, 117) and high expression of PD-L1 (122). Recently, caveolin-1, galectin-1, and SMYD3 were found to be highly expressed in CLBC (123–125).

Some studies have found that CLBC is heterogeneous, as is its immunohistochemical expression. Fougner et al. (52) believed that they listed CLBC for analysis without considering the six intrinsic subtypes. This may have masked the intrinsic characteristics of CLBC. Pommier et al. (68) divided CLBC into three subgroups (CL-1, 2, and 3) according to FGA levels, which were derived from mammary stem cells (MaSC), mature luminal cells (mature luminal, mL), and luminal precursor cells (luminal progenitor, PL), respectively, which have the characteristics of expressing stem cell-related protein markers, luminal-related markers, and basal-like markers. This is in line with the findings of Fougner et al. (52) CLBC penetrates every subtype of breast cancer. Therefore, the immunohistochemical expression of CLBC is a complicated process, and different markers are expressed depending on the origin of the tumor.

## 8 The prognosis of claudin-low breast cancer

Prat et al. (114) analyzed UNC337 and two independent gene expression databases (NKI1295 and MDACC133) with nine cell lines (claudin-low and PAM50 subtype predictors) and found that the incidence of CLBC was 7%~14%. In terms of prognosis, the Kaplan-Meier analysis of the two databases showed that the prognosis of CLBC (HR 2.83, RFS and OS 5.66) was significantly lower than that of luminal A subtype breast cancer (HR 4.71, RFS and OS 17.98). A study by Dias et al. (115) in 2016 showed that the overall survival (OR) of CLBC at 3, 5, and 10 years was higher than that of the basal-like subtype and lower than that of other subtypes, and the local recurrence rate was higher than that of other subtypes. Disease-free survival (DFS) is lower than that of luminal A breast cancer but higher than that of other subtypes. Sabatier et al. (114) found that the five-year local recurrence survival rate (67%) of CLBC is similar to that of luminal B (64%) and basal-like (60%) but higher than that of the HER2-enriched type (55%) and lower than that of luminal A (79%). Lu et al. (50) found that claudin-low has worse disease-free survival than non-CLBC, which is consistent with the conclusion found by Xu et al. (119). Overall, the prognosis for CLBC is poor.

Fougner et al. (52) conducted a study on the METABRIC database and found that, when CLBC was studied as a single group, the results showed that CLBC was associated with a poor prognosis, which was consistent with the findings mentioned above. However, when they analyzed CLBC according to the intrinsic molecular classification of breast cancer, the results showed that the basal-like Claudin-low had the worst prognosis, which was consistent with the prognostic results of the intrinsic molecular classification. In addition, the analysis of the claudin-low and non-claudin-low subtypes of the same intrinsic subtype of breast cancer showed that the difference was not statistically significant, indicating that there is no evidence that claudin-low can affect the prognosis of breast cancer.

Several studies have found that many immunohistochemical markers are helpful in evaluating the prognosis of CLBC. TBL1 is necessary for the mesenchymal phenotype of transformed breast epithelial cell lines and claudin-low subtype breast cancer cell lines. The high expression of the *TBL1* gene is related to poor prognosis and an increased metastasis rate in breast cancer patients, indicating that the expression level of TBL1 can be used as a prognostic marker (62). According to the clinical data analysis of Fenizia C et al. (125), a higher level of SMYD3 is related to the poor prognosis of CLBC and the reduction in metastasis-free survival of breast cancer patients.

## 9 The pathological response to chemotherapy and the progressive treatment of claudin-low breast cancer

At present, research on CLBC treatment is still in the basic research stage, and most of the studies have not yet entered the animal experiment and clinical trial stages. At present, studies on the pathological complete remission of claudin-low breast cancer are based on the follow-up of the original cases. Prat et al. (45) followed up 133 patients with the MADCC Breast Cancer Database and found that the complete pathological remission rate (pCR rate, 38.9%) of CLBC was significantly lower than that of the basal subtype (pCR rate, 73.3%) but higher than that of luminal A and B. Sabatier et al. (110) studied 5,447 patients, and 1,294 patients received neoadjuvant chemotherapy with a pCR rate of 23%. The pCR rate of 228 CLBC patients was 32%, which was close to that of the basal sample (pCR rate, 33%), lower than that of the HER2-rich type (pCR rates, 37%), and much higher than that of the luminal A and luminal B subtypes (pCR rates, 7% and 18%). Both results showed that the pCR rate of CLBC is higher than that of other subtypes except basal-like breast cancer, indicating that CLBC is relatively sensitive to chemotherapeutic drugs.

Immunotherapy of CLBC. Fougner et al. (52) pointed out that CLBC showed high lymphocyte infiltration and high expression of PD-L1 and T lymphocyte reaction, which may provide opportunities for immunotherapy. At present, the means of immunotherapy mainly include immune checkpoint inhibitors and anti-PD1/PD-L1 therapy, etc. CLBC is rich in lymphocyte infiltration and drinks high expression of PD-L1 and IL6. These immunotherapy can provide new treatment schemes for CLBC patients (126). There is a lack of identification of CLBC from intrinsic molecular classification of breast cancer in clinical practice, so there is little research on immunotherapy of CLBC. In addition, some researchers have found that CLBC is rich in Tregs. Depletion of Tregs and suppression of immune checkpoints can weaken tumor growth and prolong survival, but they cannot lead to tumor regression (127).

Several novel targets have been proposed for the treatment of CLBC. Chang et al. (128) found that PIK3CG is a potential target for the treatment of CLBC. Inhibiting the activation of PIK3CG can enhance the therapeutic effect of PTX on CLBC. Stalker et al. (129) discovered that PDGFR inhibitors (sunitinib, regorafenib, and masitinib) can be used to inhibit the migration and metastasis of CLBC cells. Some scholars found that the combination of pyrazole derivatives and doxorubicin can increase the death of MDA-MB-231 cells (130). Panobinostat combined with gefitinib can synergistically inhibit the proliferation of CLBC cells and promote apoptosis (131, 132). CLBC is rich in EMT features, which may provide a new therapeutic target for chemotherapy. ZEB1, AP-1, and TEAD/YAP, the effector of the HIPPO pathway, form a transactivation complex to activate oncogenes, disrupting their molecular interaction may provide a promising treatment for CLBC (133). Recently, some new therapeutic targets have been found. AgNPs can selectively induce lipid peroxidation and cause irreversible proteotoxicity in CLBCs (134). SLC20A1 siRNA knockdown leads to suppression the activity of CLBC, and high expression of SLC20A1 indicates a poor prognosis (135). Studies have identified PVT-1 as a long noncoding RNA can regulate the expression of Claudin-4 in CLBC, indicating that it may be an important target for treating CLBC (136). Although some therapeutic targets for CLBC have been discovered, there is still a long way to go before clinical application.

## 10 Conclusions and perspectives

In summary, CLBC is derived from breast cancer stem cells, luminal precursor cells, and luminal mature cells. The process of transforming to CLBC is related to EMT. We summarized the clinicopathological features of CLBC (**Table 2**), which is highly expressed in the immune response, breast cancer stem cells/tumor initiating cells, and EMT-related gene markers and is related to the MAPK pathway. CLBC is more aggressive because of its low expression of adhesion-related proteins, which show larger tumor size, a higher histological grade, and higher lymph node metastasis. The histological type is mainly invasive ductal carcinoma, and metaplastic carcinoma is associated with medullary carcinoma. CLBC is sensitive to chemotherapy, but its overall prognosis is poor. Currently, our knowledge of claudin-low breast cancer is inadequate due to the lack of uniformity in the classification of claudin-low breast cancer by previous investigators and the limited sample size of the study, thus leading to some bias in the results of the investigators. In the future, the origin of claudin-low breast cancer and the classification criteria by immunohistochemistry still need investigation. The potential therapeutic targets ans strategies for claudin-low breast cancer should be studied.

**Table 2:** The clinicopathological features of Claudin-low breast cancer.

The clinicopathological features of CLBC		
Patient age	younger than other subtypes	controversial
Tumor characteristics		
**tumor size**	larger	
**histological grade**	higher	
**lymph node metastasis**	easy	
**Histopathology**	nonspecialized type breast cancer and metaplastic carcinomas	
Immunohistochemistry		
**low expression or no expression**	claudin3, claudin4, claudin7, E-cadherin, HER-2	
**high expression**	Vimentin, SNAI1, SNAI2, TWIST1, TWIST2, ZEB1, ZEB2, ALDH1	
**Treatment**	at the basic medical research stage	
**pCR**	pCR rate of CLBC is higher than other subtypes	
**Prognosis**	poor	controversial

## Author contributions

CP and AX wrote and edited the paper, they have made the same contribution to this work and share the identity of the first author. XM, YZ and YY generated the tables and figure. CC and CW revised the paper. All authors contributed to the article and approved the submitted version.
